# Novel Superadsorbent Highly Porous Hydrogel Based on Arabic Gum and Acrylamide Grafts for Fast and Efficient Methylene Blue Removal

**DOI:** 10.3390/polym12020338

**Published:** 2020-02-05

**Authors:** Ahmed M. Elbedwehy, Ayman M. Atta

**Affiliations:** 1 Nanotechnology Center, Mansoura University, Mansoura 3551, Egypt; 2 Chemistry Department, College of Science, King Saud University, P.O. Box-2455, Riyadh 11451, Saudi Arabia

**Keywords:** arabic gum, porous, emulsion inversion, superadsorbent, hydrogel, water retention

## Abstract

Environmental pollution with dyes released from industrial effluent is one of the major and most critical problems in the world. To alleviate this issue, advanced and safe materials with fast and highly efficient dye removal should be designed. Great attention has been paid recently to hydrogels based on polysaccharides such as Arabic Gum (AG) grafted with polyacrylamide (PAM) and polyacrylic acid (PAA). These materials combine the merits of natural polymers such as biodegradability and non-toxicity with the high adsorption ability of PAM and PAA towards cationic dyes such as methylene blue (MB). Many previous works have been done to enhance three-dimensional (3D) structure and swelling ability of the graft copolymers by using a crosslinking agent or even adding nanomaterials as a filler inside the hydrogel matrix. However, these additives may negatively affect the adsorption ability, and few previous studies could reach 2000 mg/g of maximum MB capacity removal within a good period of time. In our work, we synthesized partially hydrolyzed polyacrylamide grafted Arabic gum (AG-g-PAM/PAA) to have both amide and carboxylate groups. The modified water dissolved graft product undergoes water in oil (W/O) emulsion using paraffin oil as the continuous phase and Triton X-100 as a stabilizing agent; then, the system was inversed to oil in water (O/W) emulsion by increasing the shear mixing rate and cross-linked using Epichlorohydrin (ECH). The precipitated graft product showed hierarchically interconnected micro and macropores’ sponge like shape with fast water swelling and high MB adsorption capacity (2300 mg g^−1^) after 45 min at near neutral pH conditions.

## 1. Introduction

Great interest has been paid recently to environmental issues such as wastewater containing dye pollutants. Dyes such as methylene blue (MB) are extensively used in many industries including leather, textiles, paper, and clothing [1,2,3]. Unfortunately, most of these dyes release into wastewater and persist in the environment due to their high stability to temperature, light, detergents, and chemicals [4]. For example, because of inefficient textile painting, more than 200,000 tons are lost to effluents every year, which can threaten human health and result in the demise of water ecosystems [5] due to the dyes’ high toxicity, non-biodegradability, and mutagenic ability [6]. Therefore, it is necessary to fabricate safe material with a fast and highly efficient ability to protect water resources from dyes and pollutants. As a result of many efforts, adsorption treatment for wastewater dye removal is considered to be a promising candidate strategy which has attracted the interests of many scientists in recent years [7,8,9]. Adsorbents including carbon, activated carbon, carbon nanotubes, graphene oxide, and nanocomposite hydrogels have been used for wastewater dye removal without generating secondary toxic byproducts [7,8,9,10]. Crosslinked hydrogel specifically shows high adsorption efficiency due to their high ability to absorb water without dissolving, which increases the adsorbents’ surface area for different adsorption mechanisms [11,12,13,14] like chemisorption, ion exchange, electrostatic, complexation, and hydrogen bonding interactions between adsorbents and targeted dyes. Hydrogel based on grafted polysaccharides such as gum, starch, and cellulose with polyacrylamide and polyacrylic acid is highly in demand for dye wastewater treatments due to being low-cost, eco-friendly, reusable, biodegradable, and easy to operate [15,16,17]. However, traditional uncrosslinked graft copolymerization suffering from solubility or poor three-dimensional (3D) structure which decreases its capacitance for dye adsorption (for example, Sharma et al. [18]) prepared cellulose grafted with 2-acrylamido-2-methylpropane sulfonic acid which showed maximum adsorption capacity (Q_max_) towards congo red (CR), crystal violet (CV), and malachite green (MG) dyes were 21, 53.5, and 46 mg g^−1^. Numerous efforts have been made to enhance adsorption capacity of hydrogel based on graft copolymers by using crosslinking agents [19,20] or adding nonmetals as a filler for better 3D structure and swelling ability [21,22]. However, these additives may affect the performance of the prepared graft copolymer; for example, Dai et al. [23] prepared hydrogel by graft copolymerization of acrylamide and acrylic acid onto carboxymethyl cellulose with an incorporation of graphene oxide (GO) as a filler. Results showed that with increasing GO content from 20 mg to 80 mg, the adsorption enhanced from 80 mg/g to 130 mg/g towards MB on the other side the swelling ability decreased from 75 g/g to 55 g/g. It was also found that the additional GO could decrease the size of pores inside the hydroel matrix which inhibit water swelling.

To the best of our knowledge, there are few research reports of hydrogel based on graft copolymers with Q_max_ near to 2000 mg/g for MB dye within a very long equilibrium adsorption time, which ranges from many hours to days [24,25]. On the one hand, water in oil emulsion refers to a phenomenon that occurs when water droplets dispersed in oil dissolved materials (the continuous phase) by using a suitable stabilizing agent [26], then the system undergoes phase inversion in a coagulation bath to leech out the droplets and precipitate the porous film. The previous method is extensively used in the fabrication of porous dialysis membranes for water purification, pharmaceutical products (e.g., scaffolds for drug delivery), and detergents [27,28,29]. On the other hand, emulsification inversion occurs when the dispersed phase inversed to the continuous phase or the revere [26], and this could be driven by controlling many factors such as shear rate, viscosity, and temperature. This method is highly recommended for preparing hierarchically porous materials having different pores size [30]. According to the literature review, no one has used this method for the preparation of hydrogel based on graft copolymer, and used it for MB dye adsorption. For a preliminary comparison, AG-g-PAM/PAA underwent conventional crosslinking using ECH in an alkaline environment. Both systems were used for adsorption of MB (200 mg/g) from an aqueous solution. The results revealed an ability of the novel porous hydrogel to adsorb 99% of MB dye in only 10 min however the conventional crosslinked graft copolymer didn’t show significant MB dye adsorption after 10 min.

## 2. Experimental

### 2.1. Materials

Arabic gum (BDH, Poole, Dorset, UK), acrylamide (AM), ceric ammonium nitrate (CAN), and nitric acid (AR grade) all were purchased from (Acros, Merelbeke, Belgium). Acetone and Methanol were purchased from (Fisher, Ocala, FL, USA). Paraffin oil with viscosity 100–145 centipoise (CP) at (20 °C) and Triton-X100 were purchased from (Sigma Aldrich, St. Louis, MO, USA). Doubly distilled water was used throughout the experiment. All the chemicals in the experiment were of a research-grade.

### 2.2. Techniques

#### 2.2.1. Preparation of Polyacrylamide Grafted Arabic Gum

Grafting reaction was carried out under mild conditions as reported previously [31]. Arabic gum (0.5 g) was dissolved in 5 mL of distilled water under stirring. After the gum was completely dissolved, a freshly prepared solution of CAN (0.023 mol/L) in nitric acid (0.116 mol/L) was loaded into the gum solution. Finally, AM (5.3 mol/L) was directly added under stirring. Graft copolymerization experiment was conducted in a closed Pyrex cell which was put between a pair of tube fluorescent lamps (40 W) for 1.5 h. Graft polymerization experiment was terminated by adding acetone to the reaction mixture until complete precipitation. The crude product was dried in an oven at 40 °C until a constant weight was achieved. To remove the homopolymer (polyacrylamide), the crude copolymer was washed several times using 30% v/v (methanol/water). Finally, pure graft copolymer was dried to a constant weight in an oven under 40 °C to achieve grafting percentage (GP) 274%. The grafting percentage GP% was evaluated by the following equation; GP% = (A − B) × 100/(B); where A and B are the weights of pure graft and Arabic gum, respectively.

#### 2.2.2. Partially Hydrolyzation of Graft Copolymer to Introduce Carboxylate Groups

In a round bottle flask, one gram of AG-g-PAM was dissolved in 20 mL of distilled water under vigorous stirring. After that, 2 mL of 0.5 mol/L NaOH aqueous solution was added dropwise to the previous mixture under mild stirring [32]. The reaction was performed for 2, 4, and 6 h at 50 °C. The modified graft products (S2, S4, and S6) was then precipitated in acetone followed by drying in an oven at 40 °C. Neutralization equivalent was used to determine how much amide groups are hydrolyzed to carboxylate by the following equation [32]:

Neutralization equivalent (N.E.) = {(1000x)/(yz)} (1)

where x gm of the sample of an unknown hydrolyzed graft product sample and we used fixed amount 0.2 g for each sample, y is mL required of z (N) NaOH for complete neutralization.

#### 2.2.3. Preparation of the Super-Adsorbent AG-g-PAM/PAA Hydrogel

Inverse emulsion technique was performed as follows: in a 250-mL beaker equipped with an overhead stirrer (RZR 2102, Heidolph Instruments, Schwabach, Germany) 80 mL of paraffin oil (continuous phase) was added under stirring at 600 rpm. The aqueous solution was prepared by dissolving 1 g of AG-g-PAM/PAA (S4) in 20 mL of distilled water at pH = 9 at 600 rpm until completely dissolved. Before loading into the reactor, the aqueous solution was pre-emulsified by adding (2.5% w/v) of Triton X-100 as a nonionic stabilizing agent at 1300 rpm for 10 min to ensure the adsorption of surfactant on the surface of water droplets. The mixing rate was decreased to 400 rpm on the addition of 3.5 mL of ECH dropwise up to the reaction vessel using a syringe at a rate of 3.5 mL/h. After complete addition of ECH, the reaction speed increase to 2500 rpm to subject the emulsion inversion from W/O to O/W which was monitored by viscosity increment of the reaction to form a very viscous semi-solid mixture starting after 2 h of stirring. The reaction speed was decreased after that to 600 rpm and left for an additional 4 h to assure complete inversion. Finally, the mixture was subjected to precipitation in acetone as a non-solvent for the graft copolymer and to leech out the oil droplet inside the graft matrix. The highly porous graft product was then dried in an oven under 40 °C until constant weight. For preliminary comparison, AG-g-PAM/PAA was undergoing to conventional crosslinking with ECH using the same previous reaction condition without using paraffin oil and Triton X-100.

### 2.3. Characterization of Samples

Grafted and modified grafted products were coated with gold and then subjected to visualization using scanning electron microscope (SEM) (JEOL-JSM 5300, Tokyo, Japan), operating at a typical voltage of 20 kV. IR spectrophotometer (Perkin Elmer 1430, Norwalk, CT, USA) was used for obtaining the infrared spectra (FTIR). The porosity of the AG-g-PAM/PAA hydrogel was evaluated using Adobe Photoshop CC 2019 by using the following equation [24]

R (%) = A_1_/A_0_ × 100% (2)

where (A_1_) is pore’s pixel value and (A_0_) is the total pixel area of the SEM image.

### 2.4. Water Absorption and Retention Capacities

To investigate the swelling ratio of the hydrogel, fixed weight (0.5 g) of AG-g-PAM/PAA hydrogels prepared with two different methods were immersed separately into distilled water at room temperature. The swollen hydrogels were taken out, and then the water adhered on the surface of hydrogel was carefully removed by using filter papers. The weighting process was repeated 3 times at different time interval (2–320) minutes to obtain a constant weight for each measured sample. The swelling ratio of the hydrogels was calculated by Equation (3) [33]:

SR = (Mt − M_0_)/M_0_ (3)

where M_0_ (g) is the initial weight of dry sample, and Mt (g) is the weight of swollen sample at time t (min).

The water-retention property for porous AG-g-PAM/PAA hydrogel prepared in emulsion and by the conventional method were performed at different temperatures (45 and 60) °C. The fixed weight (0.5 g) of the dried hydrogels were immersed in distilled water until maximum water absorption. After that, the swollen hydrogels were put into an oven at a different time interval (2–24) hours. The water retention ratio (WR) of the hydrogels was determined by the following equation [24]:

WR = ((Ws − W_d_)/W_d_ × 100%)/SR (4)

where Ws is the mass of the swollen hydrogel at different temperatures with a different time interval and W_d_ is the mass of the dried hydrogel.

### 2.5. Adsorption of MB Using Superadsorbent AG-g-PAM/PAA Hydrogel

The influences of initial MB concentration, initial pH values from 1.2 to 10.5, and contact time from 5 min until 2 h were all investigated. The initial pH value of the solution was tuned by using acid (HCl) or base (NaOH). All previous effects were studied using fixed weight (0.03 g) of AG-g-PAM/PAA hydrogel in closed bottles with 120 mL capacity at room temperature under shaking at 120 rpm for 2 h. After equilibrium time, the bottles were then taken out and the amount of adsorbed MB was evaluated using ultraviolet spectrophotometer (UV-1800, Shimadzu Corporation, Kyoto, Japan) at the maximum wavelength (λ_max_ = 662). The adsorption percentage (Ads. %) was determined by the following [20]:

Ads. % = (C_i_ − C_f_)/C_f_ × 100 (5)

where C_i_ is the initial concentration of MB and C_f_ is the final concentration of MB.

### 2.6. The Reusability of the AG-g-PAM/PAA Hydrogel

To study the reusability of the hydrogels for MB adsorption, a fixed weight (0.03 g) of hydrogel was added to a 100 mL MB solution (200 mg L^−1^) at pH = 7 and under room temperature. After adsorption equilibrium, hydrogels were removed from the solution by decantation. The concentration of desorbed MB was evaluated by using the UV spectrophotometer at λ_max_ = 662. The MB adsorbed onto the hydrogel then eluted under different pH values prepared from (potassium chloride/hydrochloric acid) or the pH 1–2 and (Sodium citrate/Citric acid) for the pH 3–6 and then used for the next adsorption cycle. Desorption percentage (Des. %) was determined by the following [33]:

Des. %= (C_d_/C_a_) − 100 (6)

where C_d_ is the concentration of desorbed MB by the acid and C_a_ is the concentration of adsorbed MB by using the hydrogel.

## 3. Results and Discussion

### 3.1. Preparation and Characterization of AG-g-PAM/PAA Hydrogel

**Scheme 1:**
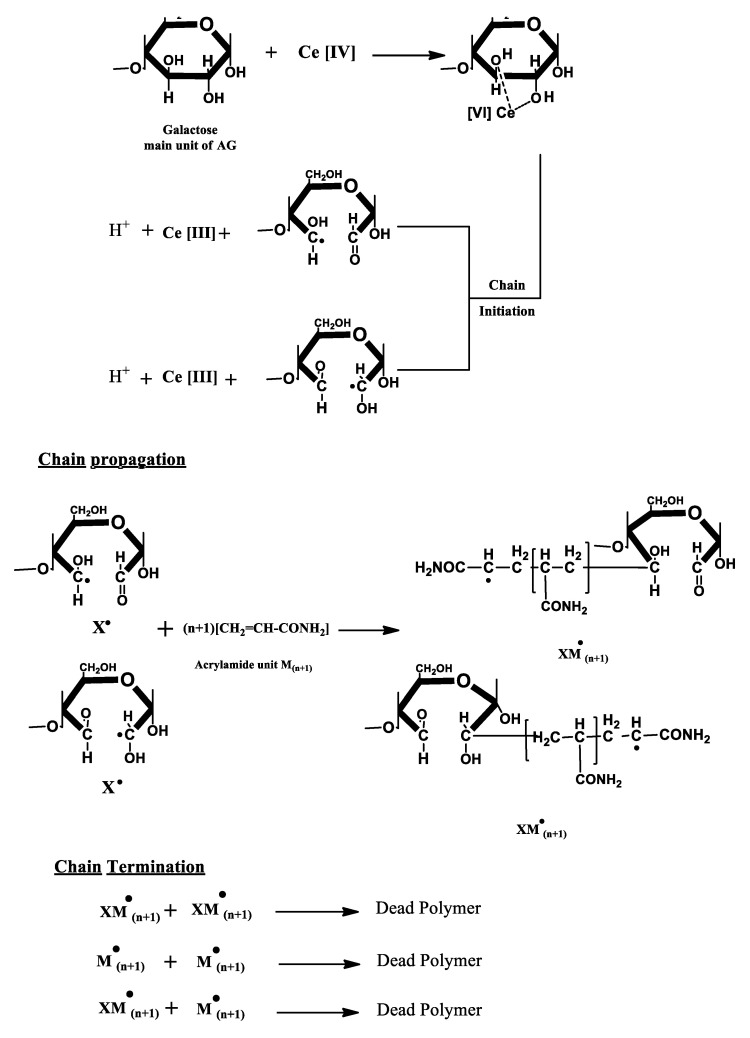
Graft Copolymerization of Acrylamide onto Arabic gum.

**Scheme 2:**
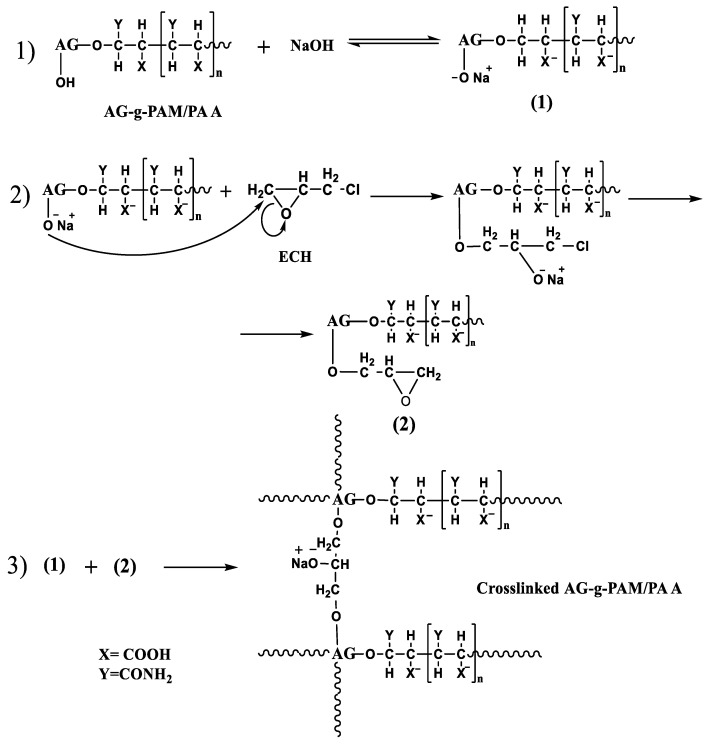
Crosslinking of AG-g-PAM/PAA.

The proposed chemical structure and the synthesis procedure for preparing PAM–g–AG are represented by Scheme 1. Crosslinking mechanisms of PAM–g–AG are also shown in Scheme 2. The mechanism by which Ce(IV) generates free radicals is based on the formation of coordination complex between Ce(IV) and the hydroxyl groups of AG, which is then disproportionate, forming a free radical on the biopolymer chain and Ce(III) [34]. The produced radicals of AG initiate the grafting of AM and PAM onto the backbone of Arabic gum. N.E. indicates the number of basicity of the partially hydrolyzed AG-g-PAM. From (Table 1) N.E., values gradually decreased with the reaction time from S2 to S6 at the same temperature (50 °C) and same NaOH concentration (0.5 mol/L). Lower N.E. values mean the greater number of carboxyl groups since one ±OH group is introduced after the replacing of NH_3_ during hydrolysis of the amide groups so the molecular weight of the polymer would remain the same [32]. S4 having moderate amide conversion to carboxylate was selected for the next crosslinking reaction.

**Figure 1:**
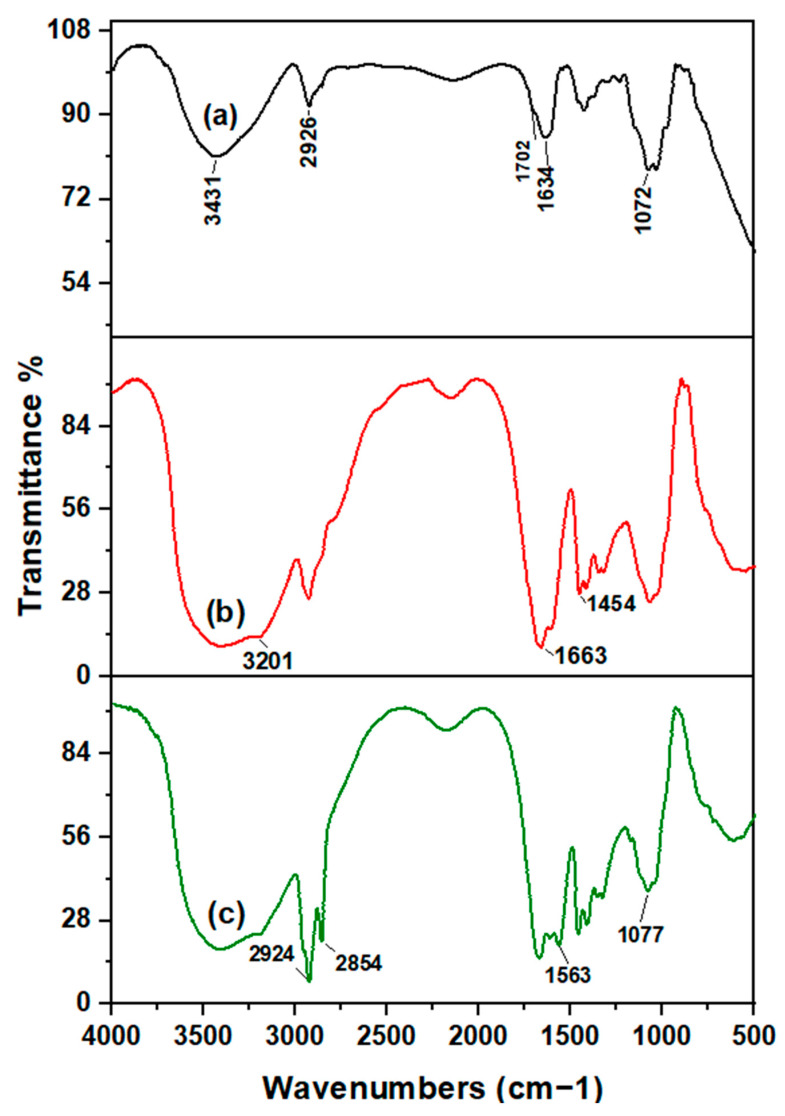
FT-IR spectra of (**a**) Acacia gum, (**b**) PAM-g-AG, and (**c**) crosslinked AG-g-PAM/PAA.

The chemical structures of original AG, PAM–g–AG, and its partially hydrolyzed structure with NaOH were elucidated from the FTIR spectra presented in Figure 1a–c. The AG spectrum (Figure 1a) showed a broad peak at 3431 cm^−1^ due to the stretching vibration of OH, and a moderate peak at 2926 cm^−1^ refers to C–H stretching vibrations [26,28]. Furthermore, a small peak at 1018 cm^−1^ and a broad peak at 1072 cm^−1^ are due to stretching vibrations of a C–O–C bond that link between different sugar units of AG [28].

On the other hand, the IR spectrum of PAM-g-AG copolymer (Figure 1b) showed a new shoulder peak at 3201 cm^−1^ and a sharp one at 1663 cm^−1^, respectively, corresponding to –NH and C=O stretching vibrations [31,33], thus confirming the grafting of acrylamide units onto AG. Crosslinked AG-g-PAM/PAA (Figure 1c) showed a new band at 1563 cm^−1^ due to carboxylate group stretching vibration results from the hydrolyzation of amide groups [32]. In addition, the intensity increscent at 2924 cm^−1^ and 2854 cm^−1^ indicates the increase of aliphatic methylene groups which were incorporated by ECH to the hydrogel structure [33]. The more intense peak at 1077 cm^−1^ represents more ether linkage (C–O–C), which was introduced by the crosslinking reaction [33].

**Figure 2:**
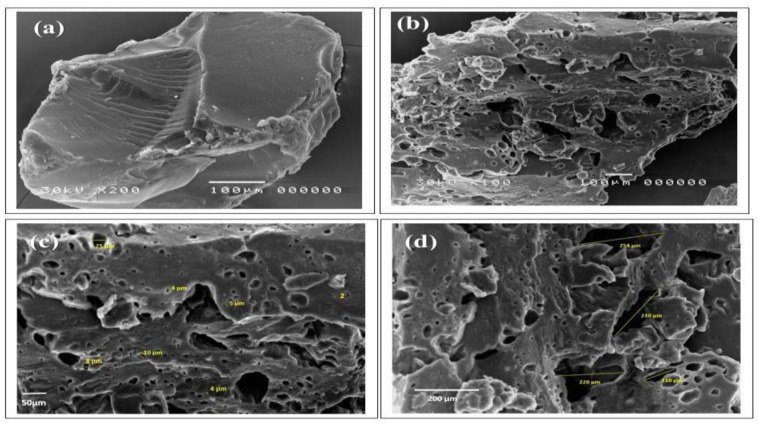
Scanning electron micrographs of (**a**) crosslinked AG-g-PAM/PAA with conventional method (**b**–**d**) crosslinked AG-g-PAM/PAA prepared in emulsion at different magnification scale.

**Scheme 3:**
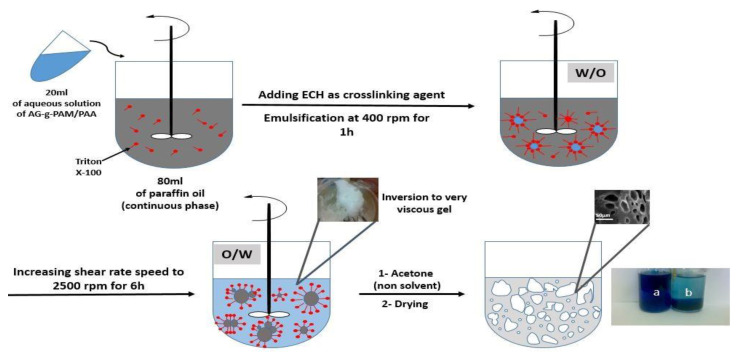
Schematic representation of crosslinked highly porous grafted gum copolymers preparation whereas the far-left side shows the adsorption of 200 ppm of MB in 100 mL distilled water after 10 min using 0.03 g of (a) crosslinked hydrogel prepared by conventional method (b) crosslinked hydrogel prepared by our method.

To confirm the formation of hydrogel pore structure, SEM images of AG-g-PAM and the AG-g-PAM/PAA hydrogel with different preparation methods are shown in Figure 2 where it can be seen that the surface of AG-g-PAM/PAA crosslinked hydrogel prepared with conventional method appeared smooth, and with no observable porosity, while the porous crosslinked hydrogel prepared in emulsion showed high pore density with macropores. The pores have a regular spherical shape and irregular shapes. The reason explains that the variety of pore size and shapes comes from the fact that during the emulsification inversion process and because of the high shear rate of mixing, the coalescence of the oil droplets could be initiated, which produces different droplet sizes and shapes (Scheme 3) [26]. The average porosity of the dried hydrogel was calculated to be 78% [24]. The high porous density with macro and micro sizes could facilitate the fast and easy penetration of dyes and water inside the hydrogel matrix, which explains the high SR of the novel porous hydrogel [24].

**Figure 3:**
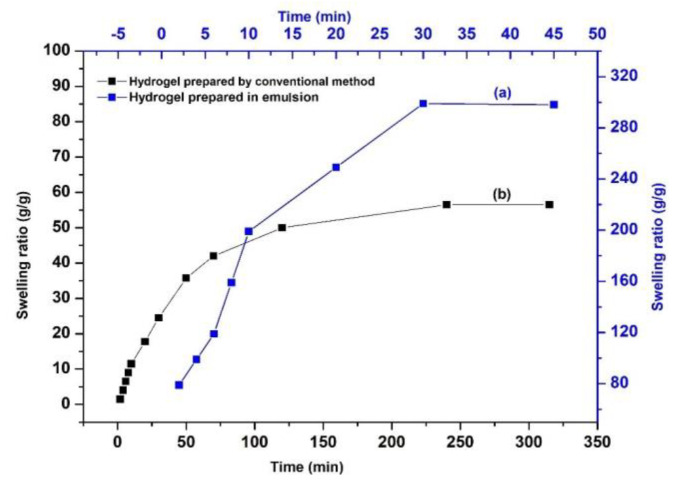
The swelling curve of (**a**) Porous AG-g-PAM/PAA hydrogel and (**b**) Conventional non-porous AG-g-PAM/PAA hydrogel in distilled water at room temperature and near-neutral pH.

The water absorption and water retention capacities of the AG-g-PAM/PAA hydrogel have a great role in the adsorption of MB dyes [20,24]. The additional swelling can increase the surface area, allowing more sites to be completely exposed to MB dyes. In our novel porous hydrogel, the 3d porous structure allows water solvent to flow simultaneously by convection and diffusion, whereas the size of the pores gradually increased, channels became wide, and the pore walls became thinner [35,36]. From (Figure 3) after 10 min, it reached 200 times its original weight and achieved equilibrium after 30 min to reach 300 times. In contrast, the nonporous hydrogel prepared by conventional method showed gradual water absorption with increasing time to reach approximately 45 times after 120 min. This great enhancement validates the role of a porous structure to enhance time and capacity of water absorption.

**Figure 4:**
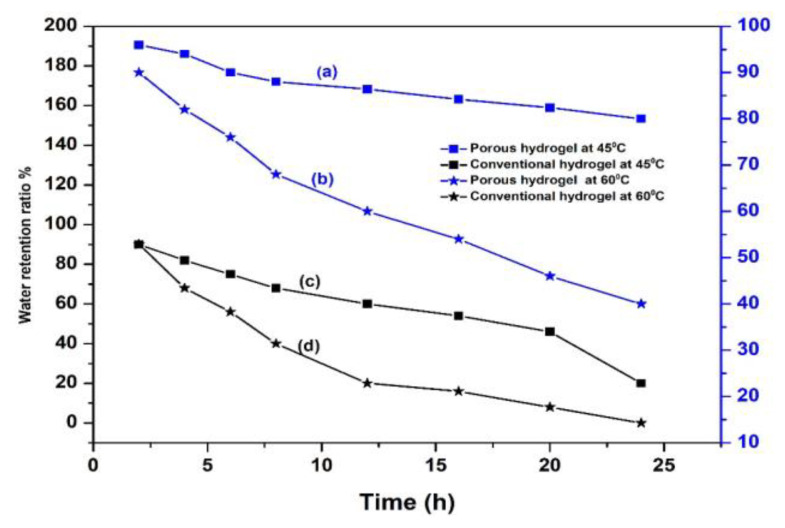
Water retention ratio% of (**a**,**b**) porous AG-g-PAM/PAM hydrogel and (**c**,**d**) Conventional non-porous AG-g-PAM/PAA hydrogel at 45 and 60 °C respectively.

Figure 4 clarifies the water retention property of the porous hydrogel at different temperatures (45 and 60 °C). In the first 4 h, the curve shows a sharp decrease, but after that, the water retention proceeds slowly. This is due to, initially, the big voids contained in the gel having a weak water connection and being easily disconnected. With the passing of the time, more small pores beside the substantial hydroxyl, amide, and carboxylate groups in the hydrogel made the interaction stronger, which enhanced the water retention, leading to a relatively slow dehydration [24,37]. It could retain approximately 80% and 40% of its weight after 24 h at 45 and 60 °C, respectively. On the other side, the conventional hydrogel lost all of its absorbed water within 12 h at 60 °C and could retain only 20% of its weight after 24 h at 45 °C. Therefore, the high water retention capacity of the porous hydrogel should enhance the MB removal.

### 3.2. Influence of MB Initial Concentrations

**Figure 5:**
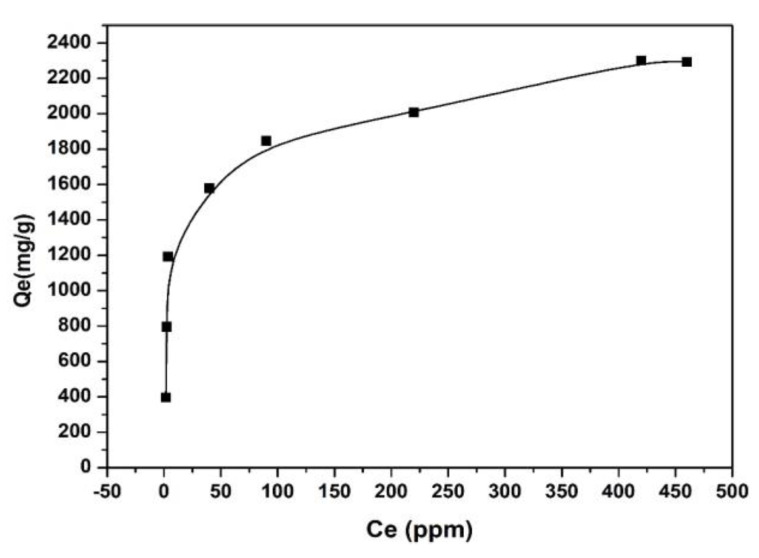
Adsorption isotherm of MB onto AG-g-PAM/PAA porous hydrogel. Adsorbent dose 30 mg/100 mL and shaking time 2 h in pH 7 and at room temperature.

The influence of the initial MB concentration on the adsorption capacities (Qe) of the porous hydrogel near-neutral pH and at room temperature was determined by changing the initial concentration of MB in the range of 200 to1600 mg/L. Data in Figure 5 showed that the low Qe values were obtained with low initial MB concentrations, due to the unsaturation of the adsorption sites provided by the hydrogel [33]. The Qe value would increase until obtaining the saturation for the active sites of the hydrogel. The maximum adsorption capacity (Q_max_) of the adsorbent for MB was 2300 mg/g. These interesting results suggest that our novel porous hydrogel could be used as an economic industrial adsorbent for MB dye adsorption.

### 3.3. Influence of pH on MB Adsorption

The influence of pH on the adsorption percentage (Ads. %) of MB by the porous hydrogel is shown in Figure S1. The data show a relative decrease in MB adsorption under both acidic and basic pH values; at acidic pH (1–5), the relative decrease in adsorption may attribute to the competition between excess H^+^ ions and dye molecules, which decreases the number of hydrogen bonds between the hydrogel and MB [24,33]. Whereas at base pH values (9–13) some of the carboxylic groups are ionized to carboxylate which destroys the hydrogen bonding interactions, the excess of sodium ions “charge screening effect” may also hinder the electrostatic attractions between the carboxylate groups of the polymer and MB [24,33]. On the other hand, under pH = 7, the moderate amounts of the carboxylate group in addition to the amide and carboxylic groups may be optimal to the electrostatic interactions and hydrogen bonds formation between the hydrogel and MB, resulting in the maximum MB adsorption capacity near pH value.

### 3.4. Influence of Contact Time

The influence of contact time was studied by changing the equilibrium time from 2 min to one hour near-neutral pH and at room temperature for MB adsorption onto the porous hydrogel. Figure S2 clearly demonstrated that after only 10 min for initial dye concentration 1600 mg/L the amount of MB adsorbed onto the porous hydrogel was nearly 1800 mg/g. After that, the adsorption rate increased gradually until equilibrium was reached after 45 min. The short time for achieving the equilibrium revealed that our porous hydrogel has a supreme adsorption efficiency for MB dye removal application [26]. The adsorption capacity of our material was compared with other adsorbents based on PAA and PAM [38,39,40,41,42,43,44] (Table S1). To the best of our knowledge, this is the fastest and most efficient adsorbent for MB ever reported.

### 3.5. Equilibrium Adsorption Investigations

**Figure 6:**
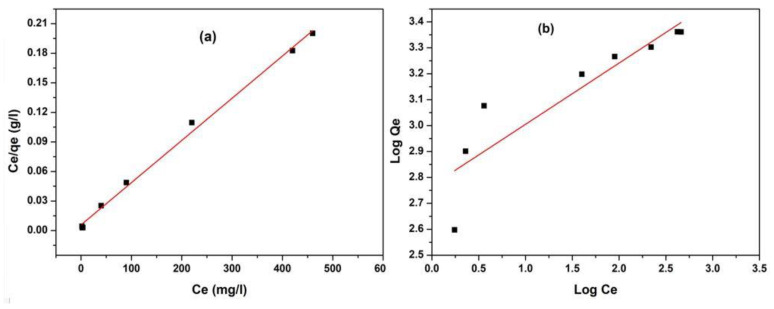
Adsorption isotherm models of MB dye on crosslinked AG–g–PAM/PAA porous hydrogel: (**a**) Langmuir and (**b**) Freundlich models.

Systems with a good adsorption design are essential for a clear understanding of the adsorption isotherms. The Langmuir (Equation (7)) and Langmuir isotherm (Equation (8)) mathematical equations were applied to explain the adsorption coverage of different ions on the solid interface to the concentration of these ions in the solution at a definite temperature [31,45].

(1/q_e_) = [(1/bq_m_) + (1/c_e_)] (7)

where b (L/mg) indicates the Langmuir equilibrium adsorption constant, and q_m_ (mg/g) is the monolayer maximum adsorption capacity. The q_m_ and b are determined by plotting C_e_/q_e_ against C_e_ (Figure 6a).

R_L_ = [1/(1 + bC_0_)] (8)

where C_0_ refers to the largest initial MB dye concentration. R_L_ is an important value to examine the type and the nature of the sorption process as the adsorption is unfavorable when R_L_ > 1, favorable when its value is between 0 to 1, linear if R_L_ = 1 and irreversible when R_L_ is zero [31]. In our work, the R_L_ value was 0.0095 which explain the sorbents favorability of MB adsorption on the superadsorbent hydrogel. Freundlich isotherm (Equation (9)) is another isothermal model which explains the adsorption on heterogeneous interfaces [24].

Log q_e_ = [(log K_F_) + (log C_e_/n)] (9)

where K_F_ (L/mg) and 1/n is the Freundlich constant, which describes the adsorption capacity and adsorption intensity. These constants are evaluated by graphing log q_e_ versus log Ce in (Figure 6b). The isotherm parameters of the MB dye adsorption are indicated in (Table 2). Results show that the Langmuir adsorption isotherm for the porous hydrogel granted the best fit (R^2^ = 0.995) compared to the Freundlich isotherm (R^2^ = 0.785). Additionally, 1/n value equal to 0.263 which indicates growing of the sorption capacity and formation of new adsorption sites [31].

### 3.6. Adsorption Kinetics

To investigate the adsorption kinetics process of MB on AG-g-PAM/PAA hydrogel, pseudo-first-order and pseudo-second-order were applied to examine the sorption kinetics data of MB dye adsorption. The pseudo-first-order Equation (10) and pseudo-second-order Equation (11) were extensively used in the kinetic studies [24,31].

**Figure 7:**
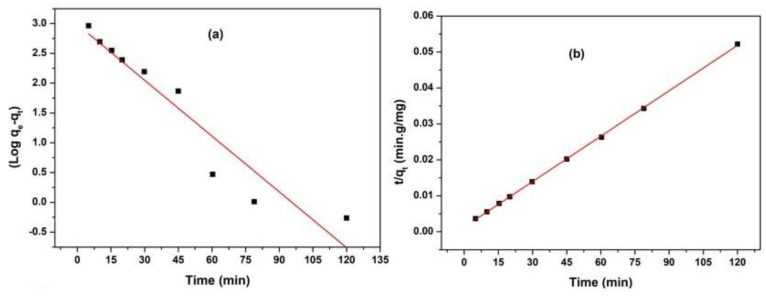
Kinetic models for adsorption of MB dye (**a**) pseudo-first order and (**b**) pseudo-second order.

Log (q_e_ − q_t_) = [(log q_e_) − (*k_i_*t/2.303)] (10)

(t/q_t_) = [(1/k_2_q_e_^2^) − (t/q_e_)] (11)

where q_t_ and q_e_ (mg/g) refer to the adsorption capacities of MB dye at time t and at equilibrium, respectively. K_1_ (min^−1^), K_2_ (g/mg min) are the rate constants at equilibrium for the pseudo-first and pseudo-second-order respectively. K_1_ constants were evaluated by plotting ln (q_e_ − q_t_) versus t in (Figure 7a) while the constants were obtained from the graphing of t/q_t_ against t in (Figure 7b) [31]. Table S2 shows the data of the rate constants and the correlation coefficients (R^2^) for both different orders for AG-g-PAM/PAA hydrogel. It could be concluded from R^2^ values that pseudo-second-order is the best fit model for our hydrogel adsorbent and its q_e_ value is in agreement with the experimental data.

### 3.7. The Reusability of the AG-g-PAM/PAA Hydrogel

The influence of various values of pH on desorption of MB by the AG-g-PAM/PAA hydrogel is shown in Table 3. The data show that the desorption percentage (Des. %) increases from 25% to 99% when changing pH values from 6 to 1. The relatively high desorption amount under strong acid treatment indicates that adsorption of MB onto AG-g-PAM/PAA hydrogel was done partially by physical attraction [24,33]. The reusability of AG-g-PAM/PAA hydrogel adsorbent was studied by repeating three cycles of the adsorption-desorption process using 0.1 M HCl. The data showed that the adsorption capacity of the AG-g-PAM/PAA could retain 98% after three cycles, as shown in Table 4. These results revealed that there was no significant loss in activity over three cycles. The interesting reusability of our novel porous hydrogel may be attributed to its cross-linked three-dimensional structure [24].

## 4. Conclusions

In summary, AG-g-PAM was prepared by free radical graft copolymerization of acrylamide onto Arabic gum. The graft product then underwent partial alkaline hydrolysis to have carboxylic groups. The water in oil emulsion inversion to oil in water for AG-g-PAM/PAA was used for creating high pore density during crosslinking reaction. The macropores channels created within the matrix structure of the novel porous hydrogel showed fast water swelling to reach 200 times its original weight after 10 min. The adsorption of MB dye onto the superadsorpent hydrogel perfectly fits the Langmuir adsorption model and pseudo-second-order models. The maximum adsorption capacity was 2300 mg/g at pH = 7 and this is the first time that PAM/PAA-based AG hydrogel adsorbent to achieve maximum adsorption capacity of more than 2000 mg/g for MB dye removal. On the other hand, the water-swollen hydrogel could retain 80% and 40% of its weight after 24 h at 45 and 60 °C, respectively, which can open a new window for release-control studies in the future.

## Figures and Tables

**Table 1:** Determination of neutralization equivalent (N.E.) of AG-g-PAM using 0.5 M of NaOH at 50 °C under different time interval (2, 4, 6) h.

Sample	Volume of 0.1 M of NaOH (V_2_) cc	V_1_^a^ − V_2_ mL	N.E. (g)
**S2**	43.6	0.6	3103.6
**S4**	42.6	1.6	1163.8
**S6**	41.7	2.5	744.8

^a^ V_1_ is the volume of 0.1 M NaOH to neutralize 40 mL of HCl = 37.7 mL.

**Table 2:** Langmuir and Freundlich isotherm constants for MB dye adsorption on crosslinked AG-g-PAM/PAA porous hydrogel.

		Langmuir Constants			Freundlich Constants		
Q_exp._	Q_m_ (mg/g)	b (L/mg)	R^2^	R_L_	K_f_	1/n	R^2^
2300	2331	0.0740	0.9958	0.0095	0.5870	0.2362	0.7855

**Table 3:** Desorption ratio of MB dye under different pH values.

pH Value	Desorption Ratio (%)
1	99
2.02	90.5
4	48.3
6.08	25.03

**Table 4:** Repeated adsorption of MB dye by AG-g-PAM/PAA porous hydrogel (initial concentration 200 mg/L, adsorbent polymer (0.3 g/L), pH 7.0, contact time 1 h and at room temperature).

Cycle Number	Desorption Ratio (%)
1	99
2	98
3	98

## Supplementary Materials

The following are available online at https://www.mdpi.com/2073-4360/12/2/338/s1, Figure S1: Influence of pH on adsorption of MB onto AG-g-PAM/PAA porous hydrogel. MB concentration 1400 mg/L, adsorbent dose 30 mg/100 mL, and shaken time 2 h at room temperature, Figure S2: Influence of time on adsorption of MB onto AG-g-PAM/PAA porous hydrogel. MB concentration 1400 mg/L, adsorbent dose 30 mg/100 mL in neutral pH and at room temperature, Table S1: Comparison of the maximum adsorption capacities of hydrogel based on PAA/PAM grafted on natural polysaccharides for the removal of cationic dyes, Table S2: Kinetic parameters for the adsorption of MB dye on crosslinked AG-g-PAM/PAA porous hydrogel.
