# Correction: Elbedwehy, A.M.; Atta, A.M. Novel Superadsorbent Highly Porous Hydrogel Based on Arabic Gum and Acrylamide Grafts for Fast and Efficient Methylene Blue Removal. *Polymers* 2020, *12*, 338

**DOI:** 10.3390/polym17131861

**Published:** 2025-07-03

**Authors:** Ahmed M. Elbedwehy, Ayman M. Atta

**Affiliations:** 1 Nanotechnology Center, Mansoura University, Mansoura 3551, Egypt; 2 Chemistry Department, College of Science, King Saud University, P.O. Box-2455, Riyadh 11451, Saudi Arabia

## Error in Figure

In the original publication [1], there was a mistake in Figure 1 as published. The authors regret the visual discrepancies in the spectral presentation of Figure 1 and sincerely apologize for this oversight and any resulting confusion it may have caused. The corrected Figure 1 appears below. The authors state that the scientific conclusions are unaffected. This correction was approved by the Academic Editor. The original publication has also been updated.

## Figures and Tables

**Figure 1:**
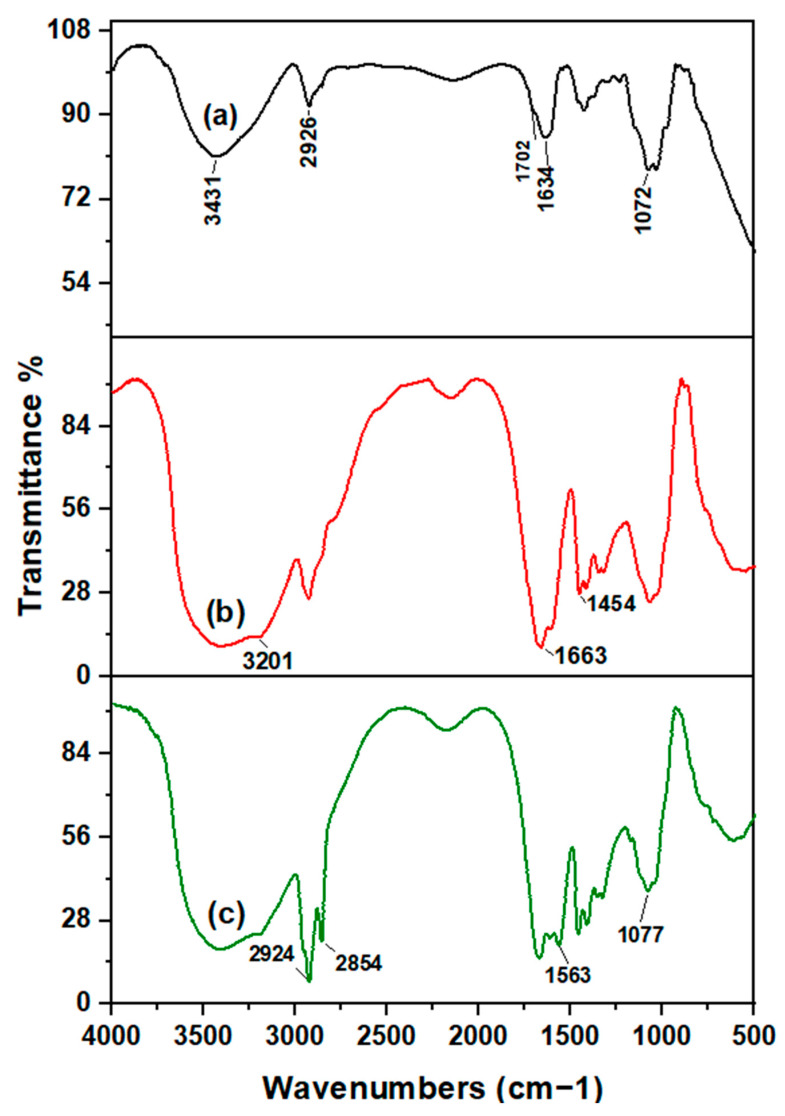
FT-IR spectra of (**a**) Acacia gum, (**b**) PAM-g-AG, and (**c**) crosslinked AG-g-PAM/PAA.
